# Long-term booster schedules with AS03_A_-adjuvanted heterologous H5N1 vaccines induces rapid and broad immune responses in Asian adults

**DOI:** 10.1186/1471-2334-14-142

**Published:** 2014-03-15

**Authors:** Paul Gillard, Daniel Wai Sing Chu, Shinn-Jang Hwang, Pan-Chyr Yang, Prasert Thongcharoen, Fong Seng Lim, Mamadou Dramé, Karl Walravens, François Roman

**Affiliations:** 1 GlaxoSmithKline Vaccines, Wavre, Belgium; 2 Department of Medicine, Queen Mary Hospital Hong Kong, Hong Kong, China; 3 Department of Family Medicine, Taipei Veterans General Hospital and National Yang-Ming University, School of Medicine, Taipei, Taiwan; 4 College of Medicine, National Taiwan University, Taipei, Taiwan; 5 Faculty of Medicine, Mahidol University, Nakon Pathom, Thailand; 6 National Healthcare Group Polyclinics, Singapore, Republic of Singapore; 7 GlaxoSmithKline Vaccines, King of Prussia, PA, USA; 8 GlaxoSmithKline Vaccines, Rixensart, Belgium

**Keywords:** H5N1, Pandemic influenza, AS03_A_-adjuvant, Prime–boost

## Abstract

**Background:**

The pandemic potential of avian influenza A/H5N1 should not be overlooked, and the continued development of vaccines against these highly pathogenic viruses is a public health priority.

**Methods:**

This open-label extension booster study followed a Phase III study of 1206 adults who had received two 3.75 μg doses of primary AS03_A_-adjuvanted or non-adjuvanted H5N1 split-virus vaccine (A/Vietnam/1194/2004; clade 1) (NCT00449670). The aim of the extension study was to evaluate different timings for heterologous AS03_A_-adjuvanted booster vaccination (A/Indonesia/5/2005; clade 2.1) given at Month 6, 12, or 36 post-primary vaccination. Immunogenicity was assessed 21 days after each booster vaccination and the persistence of immune responses against the primary vaccine strain (A/Vietnam) and the booster strain (A/Indonesia) was evaluated up to Month 48 post-primary vaccination. Reactogenicity and safety were also assessed.

**Results:**

After booster vaccination given at Month 6, HI antibody responses to primary vaccine, and booster vaccine strains were markedly higher with one dose of AS03_A_-H5N1 booster vaccine in the AS03_A_-adjuvanted primary vaccine group compared with two doses of booster vaccine in the non-adjuvanted primary vaccine group. HI antibody responses were robust against the primary and booster vaccine strains 21 days after boosting at Month 12 or 36. At Month 48, in subjects boosted at Month 6, 12, or 36, HI antibody titers of ≥1:40 against the booster strain persisted in 39.2%, 61.2%, and 95.6% of subjects, respectively. Neutralizing antibody responses and cell-mediated immune responses also showed that AS03_A_-H5N1 heterologous booster vaccination elicited robust immune responses within 21 days of boosting at Month 6, 12, or 36 post-primary vaccination. The booster vaccine was well tolerated, and no safety concerns were raised.

**Conclusions:**

In Asian adults primed with two doses of AS03_A_-adjuvanted H5N1 pandemic influenza vaccine, strong cross-clade anamnestic antibody responses were observed after one dose of AS03_A_-H5N1 heterologous booster vaccine given at Month 6, 12, or 36 after priming, suggesting that AS03_A_-adjuvanted H5N1 vaccines may provide highly flexible prime–boost schedules. Although immunogenicity decreased with time, vaccinated populations could potentially be protected for up to three years after vaccination, which is likely to far exceed the peak of the a pandemic.

## Background

The first human case of avian-origin influenza A/H5N1 infection emerged in Hong Kong in 1997, and by July 2013, the World Health Organization (WHO) had reported over 600 cases, of which more than half were lethal [1]. The majority of cases occur in people in close contact with poultry, and although H5N1 infections do not appear to transmit efficiently between humans, the pandemic potential of these viruses should not be overlooked. Indeed, the unpredictable nature of influenza viruses was recently highlighted by reports of human cases of a novel reassortant avian-origin influenza A/H7N9 strain in China [2].

The development of vaccines against potentially dangerous avian-origin influenza infections remains a public health priority, and based on circulating viruses, the WHO recommends vaccines against H5N1 should include clade 1 or clade 2 strains [3]. However, it currently takes about four to six months to produce an inactivated influenza vaccine, and the capacity of the manufacturing infrastructure is limited, meaning that mitigating the spread of infection until a vaccine becomes available and then supplying enough vaccine to meet worldwide demand are major challenges. The formulation of H5N1 influenza vaccine with the oil-in-water, tocopherol-based Adjuvant System, AS03, may help address these challenges.

In adults, AS03_A_-adjuvanted H5N1 (A/Vietnam/1194/2004) vaccine was shown to be significantly more immunogenic than non-adjuvanted vaccine, and two doses of adjuvanted vaccine containing 3.75 μg of hemagglutinin antigen (HA) fulfilled licensure criteria for vaccine immunogenicity [4]. In addition to responses against the vaccine strain (clade 1), AS03_A_-adjuvanted H5N1 vaccine provided strong cross-reactivity to clade 1, 2.1, 2.2, and 2.3 drifted strains [5]. Cross-reactive immunogenicity not only has the potential to provide protection against drift variants, but also offers the opportunity for flexible priming strategies. For example, a pre-pandemic strategy involves stockpiling vaccine matched to prevalent avian-origin H5N1 viruses based on global surveillance, to be deployed during a pandemic alert or at the start of the pandemic, which can be followed by a booster dose of vaccine matched to the actual emerging strain given when batches become available [6,7].

Previous studies of AS03_A_-adjuvanted H5N1 influenza vaccines in adults were performed in populations in Europe and North America, and included schedules with booster doses 6 or 12 months post-primary [4,8-12]. More recently, a large Phase III, lot production consistency study was conducted in adults in Taiwan, Thailand, Singapore, and Hong Kong, and confirmed that two doses of AS03_A_-adjuvanted H5N1 vaccine (A/Vietnam/1194/2004; clade 1) elicited robust hemagglutinin-inhibition (HI) and neutralizing antibody responses, which were markedly higher than those observed with two doses of non-adjuvanted H5N1 vaccine [13]. Here we describe the staggered booster extension phase of the study, in which subjects in the AS03_A_-adjuvanted H5N1 primary group received one dose of heterologous booster vaccine (A/Indonesia/5/2005; clade 2.1) at either Month 6, 12, or 36 post-primary vaccination. Subjects in the initial non-adjuvanted H5N1 primary group received two doses of AS03_A_-adjuvanted heterologous H5N1 booster vaccine at Month 6. The aims were to assess the immunogenicity, including cross-reactivity and persistence, reactogenicity, and safety of each booster vaccine schedule.

## Methods

### Design and objectives

This Phase III open-label randomized extension study of adults aged 18 to 60 years evaluated the immunogenicity and safety of AS03_A_-adjuvanted H5N1 pandemic vaccine. In the initial study, subjects received two doses given 21 days apart of primary AS03_A_-adjuvanted H5N1 pandemic vaccine or non-adjuvanted H5N1 vaccine in the control group (both A/Vietnam/1194/2004; clade 1). Data regarding the HI antibody responses to AS03_A_-H5N1 and non-adjuvanted-H5N1 primary vaccine have been previously published, including the primary endpoint of the lot-to-lot consistency of four AS03_A_-H5N1 primary vaccine lots, and immunogenicity against the vaccine and drifted strains [13].

The aim of this extension study was to assess the immune responses to heterologous booster vaccine (A/Indonesia/5/2005; clade 2.1) given at different time-points post-primary vaccination. Single doses of heterologous booster vaccine were administered to subjects in the AS03_A_-adjuvanted H5N1 primary vaccine group at either Month 6, 12, or 36 post-priming; immunogenicity against the primary vaccine strain (A/Vietnam) and the booster strain (A/Indonesia) were assessed after each booster vaccination, and the persistence of immune responses were assessed in all subjects up to Month 48 post-priming. Given their lower responses to the primary vaccination shown in the initial study, all subjects in the non-adjuvanted H5N1 primary vaccine group received two doses of booster vaccine given 21 days apart at Month 6 post-priming, and immunogenicity was assessed after each booster dose.

In the extension study, the following assessments were performed: In the AS03_A_-adjuvanted H5N1 primary vaccine group, HI, neutralizing, and cell-mediated immunity antibody responses against A/Vietnam and A/Indonesia were assessed 21 days after booster vaccine given at Month 6, 12 or 36 post-priming, and immunogenicity persistence was evaluated at Month 12, 18, 24, 30, 36, 42, and 48 post-priming, depending upon the timing of booster vaccination; in the non-adjuvanted-H5N1 primary vaccine group, HI antibody responses were assessed 21 days after each booster dose given at given at Month 6, and cell-mediated immunity antibody responses were assessed 21 days after each booster dose and at Month 12 post-priming; Reactogenicity was assessed for 7 days after each booster vaccination. Unsolicited adverse events (AEs) were assessed for 30 days after each booster vaccination, and serious adverse events (SAEs) were assessed throughout.

### Subjects and setting

This study was conducted in Taiwan, Thailand, Singapore, and Hong Kong. Subjects were eligible for inclusion if they were healthy and able to comply with the booster protocol; women of child-bearing potential had to practice adequate contraception for 30 days before each vaccination and for two months after vaccination. Exclusion criteria included: the receipt of any licensed inactivated vaccines within 2 weeks or live vaccines 4 weeks before study vaccines; previous receipt of AS03_A_-adjuvanted vaccine (exclusion criterion for initial study); previous proven contact with H5N1 wild type virus; use of immunosuppressants (for >14 days) within 6 months of study vaccines; allergy to vaccine components; pregnancy or breast-feeding; clinically significant chronic illness or acute illness at enrollment.

All participants provided written informed consent. The study protocol was approved by the Institutional Review Board of the University of Hong Kong/Hospital Authority Hong Kong West Cluster, SingHealth Centralised Institutional Review Boards, Research Ethics Committee of Taipei Veterans General Hospital, and Siriraj Institutional Review Board. The study was conducted in accordance with Good Clinical Practice and the Declaration of Helsinki. The trials for which data are reported here are registered at ClinicalTrials.gov: NCT00449670 and NCT00652743. The study protocols may be found at: http://www.gsk-clinicalstudyregister.com/ (study IDs 109630 and 111443).

### Vaccines and vaccination

The H5N1 inactivated, split-virion recombinant influenza vaccines were manufactured by GlaxoSmithKline (GSK) Vaccines (Rixensart, Belgium). The primary vaccines contained 3.75 μg HA of A/Vietnam/1194/2004, formulated with an oil-in-water based emulsion Adjuvant System (AS03) or as non-adjuvanted H5N1 vaccine. AS03_A_ contained DL-α-tocopherol (11.86 mg), squalene (10.69 mg), polysorbate (4.86 mg), and thimerosal (5 μg). The booster vaccine contained 3.75 μg HA of A/Indonesia/05/2005 formulated with AS03_A_. All vaccines were administered as a 0.5 mL dose in the deltoid muscle of the non-dominant arm.

Subjects from the initial study were invited to enter the extension phase. In the initial study, 945 and 245 subjects, respectively, received AS03_A_-H5N1 and non-adjuvanted H5N1 primary vaccine. All subjects in the non-adjuvanted H5N1 primary vaccine group who entered the extension study were allocated to receive two doses of AS03_A_-heterologous booster vaccine at Month 6. A total of 265 subjects from the AS03_A_-H5N1 primary vaccine group were randomly selected to receive one dose of AS03_A_-heterologous booster vaccine at Month 6; the remaining 578 subjects from the AS03_A_-H5N1 primary vaccine group who accepted participation in the extension trial were randomized 30%:35%:35% to receive one dose of booster vaccine at Month 12, 24, or 36. Allocation to booster group was performed by the study sponsor. Vaccination at Month 24 was put on hold due to a potential safety concern that was raised during a pooled analysis across the development programme, but which was not specifically related to this trial. The concern was subsequently lifted, and following a protocol amendment, subjects who were originally scheduled to receive booster vaccination at Month 24 instead received the booster dose at Month 36.

### Immunogenicity assessments

#### Humoral immune responses

All serological testing was performed in a central GSK Vaccines laboratory or an external laboratory using standardized, validated procedures. Blood samples were taken on Day 0, and on Days 21 and 42 (to assess priming vaccine responses), and at Month 6, Month 6 + 21 days and +42 days, at Month 12, Month 12 + 21 days, and Month 36 and Month 36 + 21 day (to assess booster vaccine responses), and at Months 18, 24, 30, 42, and 48 (to assess immune response persistence depending upon timing of booster dose). HI assays were performed using an established HI method, modified for horse rather than avian erythrocytes [14-17].

HI antibody parameters were geometric mean titers (GMT), seroprotection rates (SPR; percentage of subjects with titers ≥1:40 following vaccination), and seroconversion rates (SCR; percentage of subjects achieving an increase in titer from <1:10 pre-vaccination to ≥1:40, or at least a 4-fold post-vaccination increase in titer from a pre-vaccination titer ≥1:10). SCRs after booster vaccination (booster SCR) were based on increases in HI antibody titers from the pre-booster titer measured at the booster time-point. Subjects with a titer of ≥1:10 were considered to be seropositive.

Virus neutralization was determined using a microneutralization assay on heat-inactivated sera as described previously [14,16]. A standardized amount of virus was incubated with serial dilutions of serum, and after further incubation with Madin-Darby Canine Kidney cells, virus replication was visualized by hemagglutination of chicken erythrocytes. The neutralization titer of a serum was calculated by the method of Reed and Muench [18]. Neutralizing antibody parameters measured were GMT, seropositivity rate (percentage of subjects with titer ≥1:28), and neutralizing booster seroconversion rate (neutralizing booster SCR; percentage of subjects with pre-booster titer <1:28 and post-vaccination titer of ≥1:56, or pre-booster titer ≥1:28 and ≥4-fold post-vaccination). The assay cut-off titer was 1:28.

#### Cell-mediated immune responses

Cell-mediated immune (CMI) responses were assessed 21 days after each dose of primary and booster vaccine and at each time-point up to Month 48. Intracellular cytokine staining was used to assess CMI responses, using an adaptation of previously described methods [19-21]. Briefly, white blood cells were obtained from undiluted whole blood samples and re-stimulated *in vitro* with A/Vietnam/1194/2004 H5N1 split antigen in the presence of co-stimulatory CD28 and CD49d antibodies, and Brefeldin A. Cells were incubated with fluorescence-conjugated antibodies to surface CD4 and CD8 markers, and Th1-specific activation markers, CD40L, IL-2, TNF-α and IFN-γ. Flow cytometric acquisition was performed on a BD LSR II flow cytometer and analyzed using BD *Diva* software (BD Biosciences). Results were expressed as the frequency of CD4+ and CD8+ T-cells expressing two cytokines (doubles) or each cytokine.

### Reactogenicity and safety

Reactogenicity (solicited AEs) was assessed for 7 days after each vaccination. Subjects were given diary cards to record the occurrence and severity of injection site AEs (pain, redness, swelling, ecchymosis, induration), and general AEs (arthralgia, fatigue, fever, headache, myalgia). All solicited injection site AEs were considered to be vaccine-related, and investigators provided causality assessments for solicited general events.

Unsolicited AEs were assessed for 30 days after each after each vaccination, and SAEs were assessed throughout the extension phase. All AEs were coded by preferred term and primary system organ class using the Medical Dictionary for Regulatory Activities (MedDRA). Investigators provided causality assessments for unsolicited AEs.

### Statistical analyses

The sample sizes for the boosting cohorts were based on the assumption that at least 211 subjects would receive a booster vaccination, and if the true HI SCR observed after any booster vaccination is 60%, the probability of observing a 95% confidence interval (CI) lower limit of ≥40% is greater than 99%.

Humoral immune responses at each specified time-point were described with a 95% CI. Analyses of immunogenicity were based on the per-protocol immunogenicity cohort, including subjects who were vaccinated and for whom data were available for the outcome measure at a given time-point, without fulfilling elimination criteria (per-protocol immunogenicity cohort).

CMI responses were expressed as CD4+ or CD8+ T-cells per million T-cells. CMI responses were assessed in a subset of subjects in Taiwan (cell-mediated immunity cohort).

The incidence of reactogenicity and safety events was tabulated with a 95% CI. Reactogenicity and safety analyses were performed on the total vaccinated cohort including subjects who received ≥1 dose of vaccine, and for whom any safety data were available.

## Results

**Figure 1:**
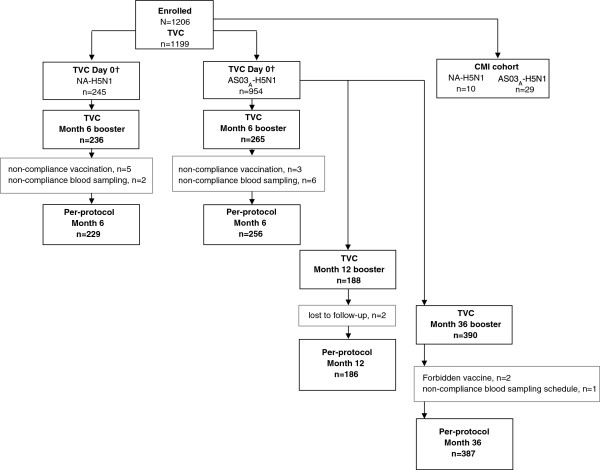
**Subject flow diagram.** †Initial study; TVC, total vaccinated cohort; CMI, cell-mediated immunity; NA H5N1, non-adjuvanted primary vaccine group’; AS03_A_-H5N1, adjuvanted primary vaccine group.

A total of 1206 subjects received primary vaccine in the initial primary vaccination study (Figure 1). In the extension study, 265 and 236 subjects from the AS03_A_-H5N1 and non-adjuvanted H5N1 primary vaccine groups, respectively, were allocated to receive booster vaccination at Month 6 (Figure 1; Table 1). The median age and standard deviations for each parameter across cohorts suggest that the groups were balanced for demographic characteristics. The mean age (range) of subjects in the per-protocol immunogenicity cohort who were boosted at Month 6 was 33.3 years (19–58 years). A total of 188 subjects received booster vaccination at Month 12 (per-protocol immunogenicity cohort), and the mean age (range) of these subjects was 35.4 years (21–59 years). A total of 387 subjects received booster vaccination at Month 36 (per-protocol immunogenicity cohort); the mean age (range) of these subjects was 38.0 years (21–62 years). Across the study groups, a limited set of 39 subjects total (17 female, 22 male) were initially enrolled in the CMI cohort in Taiwan only; the mean age (range) of subjects was 31.0 years (23–50 years). Of these, 24 subjects had available results at the final Month 48 visit.

**Table 1:** Demographic characteristics in the per-protocol immunogenicity cohort

	**Primary vaccine group**			
	**AS03**_**A**_**-H5N1**			**Non-adjuvanted-H5N1**
	**N = 954**^**†**^			**N = 245**^**†**^
	**Heterologous booster group per-protocol immunogenicity cohort**			
	**Month 6**	**Month 12**	**Month 36**	**Month 6**
	**N = 256**	**N = 186**	**N = 387**	**N = 229**
Mean age, years	33.3	35.4	38.0	33.5
(SD, range)	(10.16; 19–58)	(9.24; 21–59)	(9.74; 21–62)	(9.17; 19–58)
Male, n (%)	117 (45.7)	89 (48.4)	201 (51.9)	115 (50.2)
Female, n (%)	139 (54.3)	95 (51.6)	186 (48.1)	114 (49.8)
Central/South Asian Heritage	1 (0.4)	2 (1.1)	3 (0.8)	5 (2.2)
East Asian Heritage	154 (60.2)	115 (62.5)	225 (58.1)	135 (59.0)
South East Asian heritage	101 (39.5)	66 (35.9)	159 (41.1)	88 (38.4)
Arabic/North African Heritage	–	–	–	1 (0.4)

^†^Total vaccinated cohort in initial study; SD, standard deviation; N, number of subjects in group.

In the initial study, the first subject was enrolled in March 2007 and the last study contact was in July 2007, and in the extension study, the first subject was enrolled in September 2007 and the final visit was in June 2011.

### Humoral immunogenicity

#### HI antibody responses to heterologous booster vaccination at Month 6

**Figure 2:**
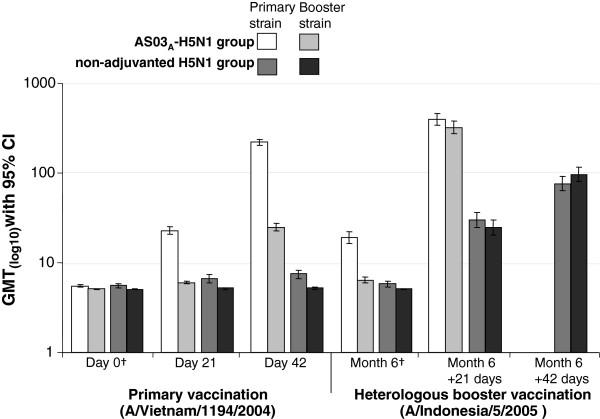
**Hemagglutination-inhibition antibody Geometric Mean Titers after primary vaccination and heterologous booster vaccination at Month 6 in the per-protocol immunogenicity cohort.** †Pre-vaccination values; CI, confidence interval; GMT, geometric mean titer. Subjects in the AS03-H5N1 primary vaccine group received one dose of heterologous booster at Month 6; Subjects in the non-adjuvanted-H5N1 primary vaccine group received two doses of heterologous booster at Month 6, given 21 days apart.

HI GMTs against primary vaccine and booster vaccine strains after the Month 6 heterologous booster vaccination are shown in Figure 2. The GMTs after one dose of heterologous booster in the AS03_A_-H5N1 primary group were 397.4 and 321.3 against the primary and booster strains, respectively, and GMTs after two doses of booster vaccine in the non-adjuvanted H5N1 primary group were 76.3 and 96.3 against the primary and booster strains, respectively (Figure 2).

**Figure 3:**
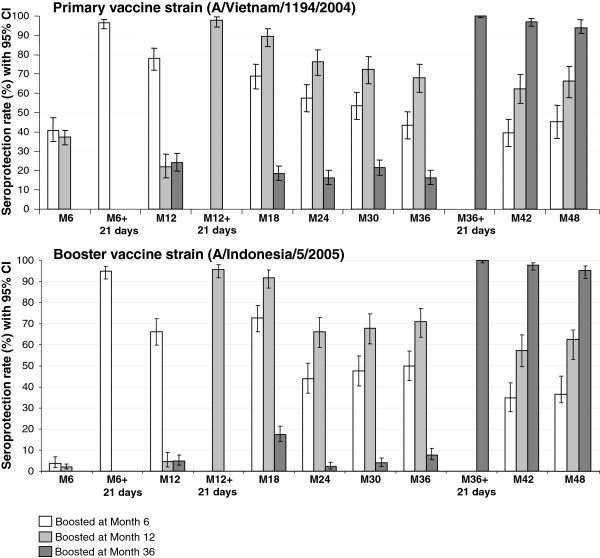
**Persistence of hemagglutination-inhibition antibody seroprotection from Month 6 to 48 in subjects who received AS03**_**A**_**-H5N1 primary vaccine in the per-protocol immunogenicity cohort.** CI, confidence interval; Seroprotection rates defined as the percentage of subjects with post-vaccination HI titers ≥1:40.

In subjects who received the AS03_A_-H5N1 primary vaccine, SCRs were 96.1% and 94.9% against the primary and booster vaccine strains, respectively, 21 days after the Month 6 booster. The booster SCRs (using Month 6 pre-booster titer as the pre-vaccination titer), against the primary and booster vaccine strains were 91.0% and 94.1%, respectively (Table 2). In subjects who received non-adjuvanted H5N1 primary vaccine, 21 days after the second dose of AS03_A_-heterologous booster at Month 6, booster SCRs were 75.9% and 82.9% against the primary and booster strains, respectively. SPRs against the primary and booster strains at Month 6 in the AS03_A_-H5N1 primary vaccine group are shown in Figure 3.

**Table 2:** Hemagglutination inhibition-based immunogenicity in the per-protocol immunogenicity cohort

**Vaccine**	**Sample time-point**	**SCR/booster SCR**^**†**^		**SPR**	
		**n/N; % (95% CI)**		**n/N; % (95% CI)**	
		**Primary strain**	**Booster strain**	**Primary strain**	**Booster strain**
**AS03**_**A**_**-H5N1**	Day 0	–	–	15/933; 1.6	1/933; 0.1
				(0.9, 2.6)	(0.0, 0.6)
	Day 21	393/925; 42.5	24/925; 2.6	412/925; 44.5	27/925; 2.9
		(39.3, 45.7)	(1.7, 3.8)	(41.3, 47.8)	(1.9, 4.2)
	Day 42	866/924; 93.7	464/924; 50.2	871/924; 94.3	464/924; 50.2
		(92.0, 95.2)	(46.9, 53.5)	(92.6, 95.7)	(46.9, 53.5)
*Heterologous booster*	Month 6	–	–	103/251; 41.0	9/251; 3.6
				(34.9, 47.4)	(1.7, 6.7)
	Month 6 + 21	233/256; 91.0^†^	241/256; 94.1^†^	242/251; 96.4	238/251; 94.8
		(86.8, 94.2)	(90.5, 96.7)	(93.3, 98.3)	(91.3, 97.2)
**Non-adjuvanted-H5N1**	Day 0	–	–	5/236; 2.1	0/236; 0.0
				(0.7, 4.9)	(0.0, 1.6)
	Day 21	8/234; 3.4	0/234; 0.0	16/234; 6.8	0/234; 0.0
		(1.5, 6.6)	(0.0, 1.6)	(4.0, 10.9)	(0.0, 1.6)
	Day 42	13/234; 5.6	1/234; 0.4	24/234; 10.3	1/234; 0.4
		(3.0, 9.3)	(0.0, 2.4)	(6.7, 14.9)	(0.0, 2.4)
*Heterologous booster*	Month 6	–	–	4/224; 1.8	0/224; 0.0
				(0.5, 4.5)	(0.0, 1.6)
	Month 6 + 21	112/228; 49.1^†^	106/228; 46.5^†^	120/223; 53.8	105/223; 47.1
		(42.5, 55.8)	(39.9, 53.2)	(47.0, 60.5)	(40.4, 53.9)
	Month 6 + 42	173/228; 75.9^†^	189/228; 82.9^†^	180/228; 78.9	189/228; 82.9
		(69.8, 81.3)	(77.4, 87.5)	(73.1, 84.1)	(77.4, 87.5)

CI, Confidence Interval; n/N, number of subjects fulfilling criteria for defined outcome/total number of subjects in group; SCR, seroconversion rates defined as percentage of subjects achieving an increase in HI titers from <1:10 to ≥1:40 or at least a 4-fold post-vaccination increase in HI titer from a pre-vaccination titer ≥1:10; ^†^Booster SCR was SCR based on increases in HI antibody titers from pre-vaccination titers at Month 6; SPR, seroprotection rate defined as the percentage of subjects with HI titers ≥1:40 following vaccination; primary vaccine strain, H5N1/A/Vietnam/1194/2004; booster strain H5N1/A/Indonesia/5/2005.

The 95% CIs for the post-booster immunogenicity parameters do not overlap and are widely separated between the AS03_A_-H5N1 and the non-adjuvanted H5N1 primary vaccine groups. This suggests that AS03_A_-H5N1 primary vaccine followed by one dose of booster vaccine provides significantly higher HI antibody responses than non-adjuvanted H5N1 primary vaccine followed by two doses of booster vaccine.

#### HI antibody responses to heterologous booster vaccination at Month 12 and Month 36

SPRs against the primary and booster strains in subjects boosted at Month 12 or 36 are shown in Figure 3.

In subjects boosted at Month 12, booster SCRs 21 days after vaccination (using Month 12 pre-booster as the pre-vaccination titer) were 95.6% and 93.9%, against the primary and booster strains, respectively.

In subjects who were not boosted at Month 6 or 12, 16.3% and 7.8% of subjects were seroprotected (titer ≥1:40) for the primary and booster strain, respectively, before the booster at Month 36. In subjects who received AS03_A_-H5N1 primary vaccine, 21 days after heterologous booster vaccination given at Month 36, the SPR was 100% against the primary and booster strains (Figure 3). Booster SCRs (using Month 36 pre-booster as pre-vaccination titer) were 99.5% and 98.1% against the primary and booster strains, respectively.

#### Persistence of HI antibody responses to heterologous booster up to Month 48

GMTs and SPRs in the per-protocol persistence cohorts are shown in Table 3 and Figure 3. In subjects who received AS03_A_-H5N1 primary vaccine followed by booster at Month 6, GMTs at Month 12 were 76.1 and 46.9 against the primary and booster strains, respectively. At Month 18, 36, and 48, SPRs were 69.0%, 43.3%, and 45.4%, respectively, against the primary vaccine strain, and 72.7%, 50.0%, and 39.2%, respectively, against the booster strain.

**Table 3:** **Persistence of hemagglutination-inhibition antibody geometric mean titers with 95**% **CI in the per-protocol immunogenicity cohort**

**Month**	**6**	**6 + 21 days**	**12**	**12 + 21 days**	**18**	**24**	**30**	**36**	**36 + 21 days**	**42**	**48**
**Boosted at Month 6**											
Primary strain	N = 256	N = 256	N = 228	–	N = 216	N = 200	N = 200	N = 208	–	N = 201	N = 194
	19.1	397.4	76.1		43.7	32.2	27.5	24.3		20.1	22.9
	(16.5, 22.5)	(342.5, 461.0)	(62.5, 92.7)		(36.7, 52.0)	(27.3, 37.9)	(23.6, 32.2)	(20.6, 28.6)		(17.3, 23.5)	(19.6, 26.9)
Booster strain	N = 256	N = 256	N = 228	–	N = 216	N = 200	N = 200	N = 208	–	N = 201	N = 194
	6.4	321.3	46.9		4.9	22.2	22.1	24.5		17.8	19.5
	(6, 6.9)	(274.3, 376.2)	(38.3, 57.4)		(45.6, 66.1)	(18.8, 26.0)	(18.8, 26.0)	(20.6, 29.1)		(15.2, 20.9)	(16.6, 23.0)
**Boosted at Month 12**											
Primary strain	N = 665	–	N = 178	N = 184	N = 182	N = 177	N = 177	N = 178	–	N = 176	N = 170
	18.5		11.8	649.7	145.2	68.5	53.0	51.2		39.0	41.8
	(5, 5.1)		(10.0, 14.0)	(549.6, 768.1)	(118.1, 178.6)	(55.9, 83.9)	(44.6, 63.1)	(42.3, 62.0)		(32.6, 46.6)	(34.7, 50.4)
Booster strain	N = 665	–	N = 178	N = 184	N = 182	N = 177	N = 177	N = 178	–	N = 176	N = 170
	6.3		6.2	338	190.7	51.3	45.5	52.4		37	37.9
	(6, 6.5)		(5.7, 6.7)	(280, 407.2)	(154.3, 235.7)	(44.6, 63.1)	(37.2, 55.8)	(42.3, 65.1)		(30.3, 45.2)	(30.9, 46.4)
**Boosted at Month 36**											
Primary strain	–	–	N = 365	–	N = 429	N = 411	N = 413	N = 387	N = 379	N = 378	N = 367
			11.3		10.2	10.8	10.7	10.5	653.1	225.4	165.1
			(10.1, 12.6)		(9.3, 11.3)	(9.8, 11.8)	(9.7, 11.7)	(9.5, 11.6)	(604.3–705.9)	(205, 247.9)	(149.8, 181.9)
Booster strain	–	–	N = 364	–	N = 429	N = 411	N = 413	N = 387	N = 379	N = 378	N = 367
			6.2		9.6	6.0	6.3	7.0	877.5	263.5	171.3
			(5.8, 6.7)		(8.7, 10.6)	(5.7, 6.3)	(6.0, 6.6)	(6.5, 7.6)	(809.1, 951.6)	(238.3, 291.3)	(155.0, 189.3)

CI, confidence interval; N, number of subjects in group; Responses are based on per-protocol immunogenicity cohort before vaccination and at vaccination + 21 days; responses at time-points post-vaccination are based on per-protocol immunogenicity persistence cohorts; Primary vaccine strain, H5N1/A/Vietnam/1194/2004; booster strain, H5N1/A/Indonesia/5/2005.

In subjects who received a booster dose at Month 12, SPRs at Month 18 were 89.6% and 91.8% against the primary vaccine and booster strains, respectively. HI antibody responses persisted until the end of follow-up (a minimum of 2 years); SPRs at Month 36 and 48 were 68.0% and 67.1%, respectively, against the primary vaccine strain, and 70.8% and 61.2%, respectively, against the booster strain.

In subjects who received a booster dose at Month 36, HI antibody responses persisted at Month 48; SPRs at Month 48 were 95.1% and 95.6% against the primary vaccine strain and the booster strain, respectively.

#### Neutralizing antibody responses to heterologous booster vaccination at Month 6, 12, and 36

Neutralizing antibody GMTs 21 days after one booster dose in the AS03_A_-H5N1 primary group were 1660.1 and 2917.5 against the vaccine and booster strains, respectively, and neutralizing GMTs after two doses of booster in the non-adjuvanted H5N1 group were 447.1 and 1075.8 against the vaccine and booster strains, respectively. The proportion of subjects with neutralizing antibody titers ≥1:28 and booster SCRs followed the same pattern as neutralizing GMTs after boosting at Month 6 (Table 4). The booster SCRs 21 days after one booster dose in the AS03_A_-H5N1 primary group were 74.2% and 88.9% against the vaccine and booster strains, respectively, and after two doses of booster vaccine in the non-adjuvanted H5N1 primary group were 91.1% and 95.7%, respectively (Table 4).

**Table 4:** **Neutralizing antibody-based immunogenicity with 95**% **CI in the per-protocol immunogenicity cohort**

	**Month**	**6**	**6 + 21 days**	**12**	**12 + 21**	**24**	**36**	**36 + 21**	**48**
**Titre ≥1:28 n/N; % (95% CI)**	**Booster at Month 6**								
	Primary strain	162/163; 99.4	163/163; 100	75/75; 100	–	74/75; 98.7	71/75; 94.7	–	73/75; 97.3
		(96.6, 100)	(97.8, 100)	(95.2, 100)		(92.8, 100)	(86.9, 98.5)		(90.7, 99.7)
	Booster strain	160/162; 98.8	162/162; 100	74/74; 100	–	70/74; 94.6	66/75; 88.0	–	67/75; 89.3
		(95.6, 99.9)	(97.7, 100)	(95.1, 100)		(86.7, 98.5)	(78.4, 94.4)		(80.1, 95.3)
	**Booster Month 12**								
	Primary strain	–	–	162/164; 98.8	170/170; 100	75/75; 100	73/74; 98.6	–	74/75; 98.7
				(95.7, 99.9)	(97.9, 100)	(95.2, 100)	(92.7, 100)		(92.8, 100)
	Booster strain	–	–	135/164; 82.3	170/170; 100	72/75; 96.0	69/74; 93.2	–	73/75; 97.3
				(75.6, 87.8)	(97.9, 100)	(88.8, 99.2)	(84.9, 97.8)		(90.7, 99.7)
	**Booster Month 36**								
	Primary strain	–	–	–	–	58/75; 77.3	55/75; 73.3	74/74; 100	74/75; 98.7
						(66.2, 86.2)	(61.9, 82.9)	(95.1, 100)	(92.8, 100)
	Booster strain	–	–	–	–	17/75; 22.7	19/75; 25.3	75/75; 100	74/75; 98.7
						(13.8, 33.8)	(16.0, 36.7)	(95.2, 100)	(92.8, 100)
	**Month**	**6**	**6 + 21 days**	**12**	**12 + 21**	**24**	**36**	**36 + 21**	**48**
**GMT value (95% CI)**	**Booster at Month 6**								
	Primary strain	169.9;	1566.3;	159.4;	–	74.0;	62.0;	–	71.4;
		(152.3, 189.4)	(1342.1, 1827.9)	(128.1, 198.2)		(62.5, 87.5)	(52.1, 73.7)		(61.8, 82.5)
	Booster strain	179.7;	2702.0;	134.0;	–	66.3;	55.4;	–	54.5;
		(161.8, 199.7)	(2270.4, 3215.6)	(107.4, 167.3)		(54.5, 80.8)	(45.5, 67.6)		(45.8, 64.7)
	**Booster Month 12**								
	Primary strain	–	–	204.1;	2734.9;	164.9;	121.1;	–	117.4;
				(179.9, 231.6)	(2334.4, 3204.2)	(125.7, 216.2)	(91.7, 159.9)		(92.7, 148.6)
	Booster strain	–	–	58.5;	2363.2;	148.9;	106.9;	–	103.4;
				(50.8, 67.3)	(2037.5, 2740.9)	(111.8, 198.3)	(81.6, 140.0)		(79.5, 134.4)
	**Booster Month 36**								
	Primary strain	–	–	–	–	33.3;	31.8;	1373.4;	429.0;
						(28.8, 38.4)	(27.3, 36.9)	(1023.7, 1842.6)	(322.2, 571.2)
	Booster strain	–	–	–	–	16.7;	17.3;	1154.3;	320.4;
						(15.4, 18.1)	(15.8, 19.0)	(847.8 1571.4)	(243.7, 421.4)
	**Month**	**6**	**6 + 21 days**	**12**	**12 + 21**	**24**	**36**	**36 + 21**	**48**
**Booster SCR n/N;% (95% CI)**	**Booster at Month 6**								
	Primary strain	–	121/163; 74.2	7/75; 9.3	–	1/75; 1.3	0/75; 0.0	–	0/75; 0.0
			(66.8, 80.8)	(3.8, 18.3)		(0.0, 7.2)	(0.0, 4.8)		(0.0, 4.8)
	Booster strain	–	144/162; 88.9	4/74; 5.4	–	0/74; 0.0	0/75; 0.0	–	1/75; 1.3
			(83.0, 93.3)	(1.5, 13.3)		(0.0, 4.9)	(0.0, 4.8)		(0.0, 7.2)
	**Booster Month 12**								
	Primary strain	–	–	–	141/164; 86.0	7/75; 9.3	5/74; 6.8	–	2/75; 2.7
					(79.7, 90.9)	(3.8, 18.3)	(2.2, 15.1)		(0.3, 9.3)
	Booster strain	–	–	–	159/164; 97.0	34/75; 45.3	27/74; 36.5	–	27/75; 36.0
					(93.0, 99.0)	(33.8, 57.3)	(25.6, 48.5)		(25.2, 47.9)
	**Booster Month 36**								
	Primary strain	–	–	–	–	–	–	73/74; 98.6	64/75; 85.3
								(92.7, 100)	(75.3, 92.4)
	Booster strain	–	–	–	–	–	–	75/75; 100	72/75; 96.0
								(95.2, 100)	(88.8, 99.2)

CI, confidence interval; n/N, number of subjects fulfilling definition of outcome/total number of subjects in group; GMT, geometric mean titer; Booster SCR, Booster seroconversion rate defined as percentage of subjects with pre-booster titer <1:28 and post-booster titer of ≥1:56, or pre-booster titer ≥1:28 and ≥4-fold post-booster; Primary vaccine strain, H5N1/A/Vietnam/1194/2004; heterologous booster strain H5N1/A/Indonesia/5/2005.

#### Persistence of neutralizing antibody responses up to Month 48

The persistence of neutralizing antibody responses up to Month 48 after booster vaccination in subjects in the AS03_A_-H5N1 primary group who were boosted at Month 6, 12, or 36 is shown in Table 4. The booster SCRs against the primary vaccine strain at 21 days after booster vaccination were 74.2%, 86.0%, and 98.6%, in subjects boosted at Month 6, 12, or 36, respectively, and against the booster vaccine strain were 88.9%, 97.0%, and 100%, respectively. At Month 48, the booster SCRs in subjects boosted at Month 6, 12, or 36 were 0%, 2.7%, and 85.3%, respectively, against the primary vaccine strain, and were 1.3%, 36.0%, and 96.0%, respectively, against the booster vaccine strain (Table 4).

### Cell-mediated immunity

**Figure 4:**
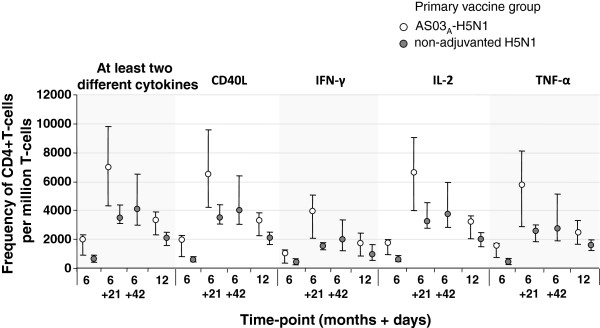
**Cell-mediated immune responses against the primary vaccine strain (A/Vietnam/1194/2004) after heterologous booster vaccine at Month 6 and persistence of responses at Month 12 (cell-mediated immunity subset).** Data expressed as median values with first and third quartiles. At Month 6, subjects in the AS03_A_-H5N1 primary group received one dose of booster vaccine, and subjects in the non-adjuvanted H5N1 primary group received two doses of booster vaccine.

No baseline CMI values were available due to a problem with the Day 0 sample shipment. At 21 days after primary vaccination, there was a substantial CD4+ T-cell response in the AS03_A_-H5N1 group. After heterologous booster vaccination at Month 6, there was a substantial CD4+ T-cell response against the primary vaccine strain. CD4+ T-cell responses persisted in both groups at Month 12 (Figure 4).

The CD4+ T-cell response in the AS03_A_-H5N1 group boosted at Month 6, 12, and 36 with follow-up to Month 48 is shown in Additional file 1. At 21 days after each booster vaccination, there was a substantial CD4+ T-cell response against the primary and booster vaccine strains. For example, 21 days after booster vaccine at Month 6, 12, and 36, median frequencies of CD40L-positive cells responsive to the booster and primary vaccine strains were 5292.0 and 6931.0, 9416.0 and 9587.0, and 5038.0 and 5296.0 per million CD4+ T-cells, respectively. Median CD40L-positive cells at Month 48 in subjects boosted at Month 6, 12, and 36 for the booster strain were 1496.0, 2013.5, and 2008.0 per million CD4+ T-cells, respectively.

There were no CD8+ T-cell responses observed in either vaccine group at any time-point.

### Reactogenicity

#### Solicited injection site adverse events

**Figure 5:**
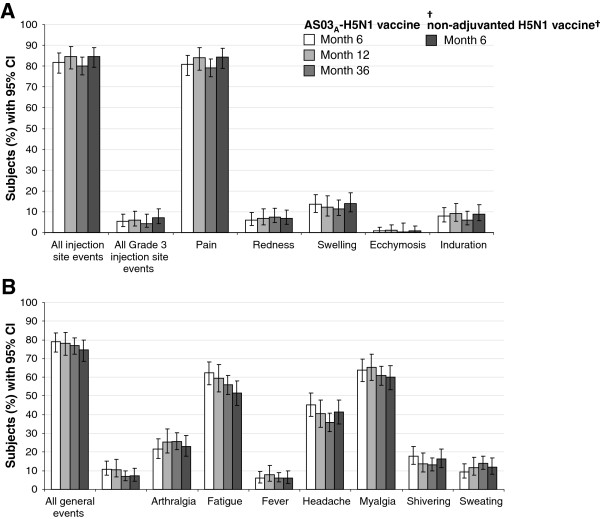
**Injection site (A) and general (B) solicited adverse events during the 7-day post-vaccination period after booster vaccination in the total vaccinated cohort.** †Subjects received two booster doses, solicited events given as overall/subject. Fever, axillary temperature >38.0°C; CI, confidence interval.

A summary of solicited injection site AEs after booster vaccination is shown in Figure 5. In the AS03_A_-H5N1 group, after booster vaccination at Month 6, 12, and 36 overall, the rate of injection site AEs was 81.9% (217/265), 84.6% (159/188), and 80.0% (312/390), respectively, including 5.3% (14/265), 5.9% (11/188), and 4.4% (17/390) Grade 3 events, respectively.

After booster vaccination, pain was the most frequent injection site event, and Grade 3 pain was uncommon. After booster vaccination at Month 6, pain was the most frequent solicited injection site AE in the AS03_A_-H5N1 primary group (80.8%; 214/265) and in the non-adjuvanted H5N1 primary group (84.3%; 198/235). There were no reports of Grade 3 pain at Month 6. After booster vaccination at Month 12, injection site pain was the most frequent solicited injection site AE (84.0%; 158/188) and Grade 3 pain was uncommon (5.9%; 11/188). After the Month 36 booster, pain was the most frequent solicited injection site AE (79.3%; 306/380). There were no reports of Grade 3 pain at Month 36.

#### Solicited general adverse events

A summary of solicited general events after booster vaccination is shown in Figure 5. In the AS03_A_-H5N1 group, after booster at Month 6, 12, and 36, the rate of general events was 78.9% (209/265), 78.2% (147/188), and 76.9% (300/390), respectively, including 10.9% (29/265), 10.6% (20/188), and 6.9% (27/390), Grade 3 AEs overall.

After booster vaccination, myalgia and fatigue were the most frequent general events, and Grade 3 general events were uncommon. In the AS03_A_-H5N1 primary group, after booster vaccination at Month 6, the most frequent solicited general AEs were myalgia (63.8%; 169/265) and fatigue (62.3%; 165/265). In the non-adjuvanted H5N1 primary group, after heterologous booster vaccinations at Month 6, the most frequent solicited general AEs overall by subject were myalgia (60.0%; 141/235) and fatigue (51.5%; 121/235). After booster vaccination at Month 12, the most frequent solicited general AEs were myalgia (65.4%; 123/188) and fatigue (59.6%; 112/188). After the Month 36 booster, myalgia (60.9%; 235/386) and fatigue (56.0%; 216/386) were the most frequent general AEs.

### Safety

#### Unsolicited AEs during the 30-day post-vaccination period

A summary of safety is shown in Table 5. During 30 days after heterologous booster vaccination at Month 6 in the AS03_A_-H5N1 primary vaccine group, there were 24.5% (65/265) unsolicited AEs, and during the 30 day post-vaccination periods after two doses of heterologous booster vaccination in the non-adjuvanted H5N1 primary group, there were 22.9% (54/236) unsolicited AEs. For 30 days post-booster dose(s) at Month 6, the most frequent AE in the AS03_A_-H5N1 and the non-adjuvanted H5N1 primary vaccine groups was nasopharyngitis (n = 8 and n = 10, respectively). After boosting at Month 12, 23.4% (44/188) of subjects reported at least one unsolicited AE. All MedDRA preferred terms were reported by no more than three subjects (1.6%), apart from lymphadenopathy (n = 7) and axillary pain and nasopharyngitis (n = 4 each). After boosting at Month 36, 21.8% (85/390) of subjects reported at least one unsolicited AE, which was most commonly influenza-like illness (n = 9).

**Table 5:** Summary of 30-day post-vaccination safety in the total vaccinated cohort

	**Primary vaccine group**	
	**AS03**_**A**_**-H5N1**	**Non-adjuvanted-H5N1**
**Month 6 booster**	**N = 265**	**N = 236**
No. subjects with ≥1 unsolicited symptom	65 (24.5%)	54 (22.9%)
No. of events by MedDRA preferred term^‡^	88	78
No. of Grade 3 events by MedDRA preferred term^‡^	6	6
No. of events by MedDRA preferred term related to vaccination^†^	26	18
**Month 12 booster**	**N = 188**	_
No. subjects with ≥1 unsolicited symptom	44 (23.4%)	_
No. of events by MedDRA preferred term^‡^	67	_
No. of Grade 3 events by MedDRA preferred term^‡^	8	_
No. of events by MedDRA preferred term related to vaccination^†^	25	_
**Month 36 booster**	**N = 390**	_
No. subjects with ≥1 unsolicited symptom	85 (21.8%)	–
No. subjects with Grade 3 unsolicited symptom	9 (2.3%)	_
No. subjects with unsolicited symptom related to vaccination^†^	34 (8.7%)	_

^†^Based on investigators’ assessment of causality; ^‡^Symptoms reported by participant after a given dose with the same preferred term were counted once; N, number of subjects in group; Subjects in the AS03-H5N1 primary vaccine group received one booster dose at allocated time-point, and subjects in the non-adjuvanted H5N1 primary group received two doses of booster vaccine at Month 6; primary vaccine strain, H5N1/A/Vietnam/1194/2004; booster strain H5N1/A/Indonesia/5/2005.

#### Serious adverse events

In subjects boosted at Month 6 in the AS03_A_-H5N1 primary vaccine group, from booster to Month 12, 1.1% (3/265) of subjects reported 5 SAEs, which were ileus, tooth disorder, tooth impacted, acute tonsillitis, and acute pyelonephritis. In subjects in the non-adjuvanted H5N1 primary vaccine group who received booster vaccinations, 2.1% (5/236) of subjects reported 6 SAEs from booster to Month 12, which were colonic polyp, *Helicobacter* gastritis, clavicle fracture, ligament rupture, and benign ovarian tumor. In subjects in the adjuvanted H5N1 primary vaccine group who did not receive booster vaccination, from Month 6 to 12, 2.2% (15/672) of subjects reported 22 SAEs. None of these SAEs were considered by the investigator to be related to vaccination.

From Month 12 to Month 12 + 30 days, 0.5% (1/188) of subjects boosted at Month 12 reported an SAE (facial bones fracture) and 0.1% (1/645) of non-boosted subjects reported 1 SAE (appendicitis). Between Month 12 and 18, 1.8% (12/645) of non-boosted subjects experienced at least one SAE, of which two were fatal (anoxic encephalopathy and acute renal failure). None of the SAEs were considered to be related to vaccination. Between Months 18 and 30, 3.8% (7/186) of subjects who were boosted at Month 12 reported 8 SAEs, and 2.5% (16/645) of subjects not boosted at Month 12 reported 24 SAEs.

In subjects boosted at Month 36, from Month 30 to 42, 3.1% (12/390) of subjects reported 15 SAEs, of which 1 (joint dislocation) resulted in withdrawal from the study. Among subjects not boosted at Month 36, 2.9% (12/401) reported 16 SAEs from Month 30 to 42, of which one was fatal (stab wound). All SAEs in subjects boosted and not boosted at Month 36 required hospitalization, and none were considered to be related to vaccination.

From Month 12 to 48, in subjects boosted at Month 6, 12, or 36 in the total vaccinated cohort, 7.1% (14/196), 7.6% (13/170), and 7.6% (28/367) of subjects, respectively, reported at least 1 SAE.

One subject who received primary vaccination but not booster vaccination experienced a pulmonary embolism three years after the second dose of primary vaccine. Blood tests revealed a low Protein S level. The investigator considered the SAE to be related to the study vaccine; despite the large interval between vaccination and the event, there was no solid evidence to exclude a causal relationship. The patient recovered upon treatment.

## Discussion

This booster study in Asian adults aged 18 to 60 years showed that one 3.75 μg HA dose of heterologous AS03_A_-H5N1 booster vaccine given at Month 6, 12, or 36 after two primary doses of AS03_A_-adjuvanted H5N1 primary vaccine, elicited robust HI antibody responses against both the booster and primary vaccine strains. Responses to heterologous booster vaccination given at Month 6 were markedly lower in subjects who were primed with non-adjuvanted versus AS03-adjuvanted H5N1 vaccine, despite receiving two versus one dose(s) of adjuvanted booster vaccine, respectively. Injection site reactions and solicited general symptoms after each booster dose were consistent with previous reports of AS03_A_-H5N1 vaccines, and no safety concerns were identified over the 48 month follow-up period.

Highly pathogenic avian-origin H5N1 influenza A viruses are endemic in domestic birds in many Asian countries, and since two human cases of H5N1 were reported in Hong Kong in February 2003, avian-origin influenza A viruses have continued to spread across Asia, Europe, and Africa [3]. The majority of H5N1 cases reported in humans since 2003 have been classified as clade 1 or clade 2, with multiple clade 2 subclades emerging (e.g. 2.1 to 2.5, of which 2.1 and 2.3 are further classified as 2.1.1 to 2.1.3 and 2.3.1 to 2.3.4) [3]. The vast majority of the 633 human cases of avian-origin influenza reported by the WHO between 2003 and July 2013 [1], were associated with bird to human transmission, and although H5N1 viruses do not transmit efficiently between humans, there have been reports of human-to-human transmission in people who have had sustained, close, and unprotected contact with infected patients [22]. Indeed, the evolution of H5N1 viruses capable of human-to-human transmission remains a threat, and as such, pandemic preparedness is paramount. In this study, we provide evidence of robust, antigen-sparing, cross-clade immune responses following AS03_A_-H5N1 pandemic influenza vaccination in a population in Taiwan, Thailand, Singapore, and Hong Kong.

In the initial observer-blind study, 21 days after a second dose of AS03_A_-H5N1 influenza vaccine, HI antibody responses fulfilled the United States Center for Biologics Evaluation and Research (CBER) licensure criteria for the vaccine strain (A/Vietnam/1194/2004; clade 1), but not a drifted strain (A/Indonesia/5/2005; clade 2.1), although substantial HI and neutralizing SCRs against the drifted strain were observed (50.2% and 91.4%, respectively) [13]. By comparison, the HI antibody responses after two doses of non-adjuvanted H5N1 vaccine did not fulfill licensure criteria for immunogenicity against either the vaccine or drifted strain [13]. These findings support previous results in a European population of adults, which showed that AS03_A_-adjuvantation enhanced immunogenicity compared with a non-adjuvanted vaccine, and demonstrated that two 3.75 μg HA doses of AS03_A_-H5N1 (*Prepandrix™*) provided robust vaccine-matched and cross-clade immune responses [4,5].

Antigen sparing vaccine formulations will maximize the number of doses available within the existing influenza vaccine infrastructure, yet this is only part of the challenge of providing adequate and rapid vaccine coverage in the event of the emergence and rapid global spread of a highly pathogenic H5N1 virus. The stockpiling of H5N1 pandemic influenza vaccines against clade 1 and clade 2 viruses to be deployed at the start of a pandemic may provide cross-protection against the emerging pandemic strain and prime a population in advance of the manufacture of a vaccine against the emerging strain [6,7]. A previous study of AS03_A_-H5N1 vaccines in adults aged 18 to 60 years evaluated various vaccination schedules and established the feasibility of the prime–boost strategy; subjects received one dose of A/Vietnam/1194/2004 primary vaccine or two doses given 21 days apart, followed by a dose of A/Indonesia/5/2005 booster or another dose of A/Vietnam/1194/2004 vaccine given at 6 or 12 months after priming. The results showed that two doses of A/Vietnam/1194/2004 vaccine given 6 months apart provided similar immune responses against the vaccine strain and higher cross-clade immune responses compared with two doses of primary vaccine given 21 days apart [12]. In subjects receiving one dose of primary vaccine, the heterologous booster at 12 months elicited robust immune responses against the primary and booster strains [12].

To further explore the flexibility of the time interval between priming and booster vaccine, we assessed a two-dose primary series of AS03_A_-H5N1 or non-adjuvanted H5N1 vaccine, followed by one dose of heterologous AS03_A_-H5N1 vaccine at Month 6, 12, or 36 in subjects in the AS03_A_-H5N1 primary vaccine group, or two doses of booster vaccine at Month 6 in the non-adjuvanted H5N1 primary group. In subjects who were primed with the non-adjuvanted vaccine, two doses of the adjuvanted heterologous booster vaccine elicited strong HI antibody responses against the primary and booster strain which fulfilled CBER licensure criteria after the second but not the first booster dose. In subjects who received AS03_A_-H5N1 primary vaccine, only one dose of booster at Month 6 months was needed to elicit booster responses that fulfilled CBER licensure criteria. Neutralizing antibody responses in the AS03_A_-H5N1 primary group supported the HI results, with a neutralizing booster SCR of 90.2% against the booster strain 21 days after the Month 6 booster dose.

Of note, HI antibody SCRs, SPRs, and GMTs after two doses of booster vaccine at Month 6 were lower in the non-adjuvanted H5N1 primary group compared with one dose of booster vaccine in the AS03_A_-H5N1 primary group. This phenomenon has been previously reported in a Phase II study which showed that HI antibody responses where markedly higher in subjects primed with AS03_A_-H5N1 vaccine compared with those primed with non-adjuvanted H5N1 vaccine [23]. Moreover, the Phase II study showed that HI antibody responses after AS03_A_-H5N1 booster vaccine were stronger in un-primed subjects compared with subjects who had received non-adjuvanted H5N1 priming vaccine, which not only highlights the role of the adjuvant for enhancing the development of immune memory, but also suggests that non-adjuvanted vaccine may have a negative effect on priming [23]. Consistent with this hypothesis are the findings from a study conducted during the 2009 swine-origin H1N1 influenza pandemic, which showed that HI antibody responses to AS03_A_-H1N1 vaccine were lower in subjects who had received a non-adjuvanted seasonal influenza vaccine during the preceding two seasons compared with those who had not [24]. The apparent negative effect of previous non-adjuvanted seasonal influenza vaccination on responses to AS03_A_-H1N1 vaccination did not appear to be associated with HI antibody levels at baseline, and the cause of this phenomenon remains to be established [24].

This study also demonstrated the feasibility of a highly flexible booster schedule, with strong cross-clade anamnestic responses observed with the booster dose given at Month 6, 12, or 36 post-priming. HI antibody responses fulfilled CBER licensure criteria against the primary and booster strain after booster vaccination at Month 6, 12, and 36, and GMTs after booster vaccination appeared higher than those observed after primary vaccination, suggesting strong immune memory recall. Priming with AS03_A_-adjuvanted pre-pandemic vaccine induced immune memory, which will allow for low-dose, rapid cross-protection with boosting at various time-points, offering highly flexible heterologous prime–boost schedules that can be adapted according to global need, pandemic strain evolution, vaccine circulation, and logistical implications in the event of a H5N1 pandemic outbreak. This should help mitigate transmission and reduce the severity of disease during the different phases of a pandemic.

To evaluate the level of protection offered after priming and boosting, we assessed immune persistence at various time-points up to 48 months post-primary vaccination. At Month 48, HI SPRs against the booster strain were 39.2%, 61.2%, and 95.6% in subjects boosted at Month 6, 12, or 36 post-priming, respectively. Although immune responses decreased with time, this result suggests that vaccinated populations could be potentially protected for up to three years after primary or booster vaccination, which is likely to far exceed the peak of the pandemic.

The induction of T and B cells is an important component of the immune response, and CD4+ effector and memory T cells have various protective roles in the innate response to influenza infection [25]. AS03_A_-adjuvanted H5N1 pandemic influenza vaccine has been previously shown to elicit strong T-cell and B-cell responses in adults, which indicates immune memory and antibody persistence, arising from both quantitative and qualitative changes in T-cell responses [26]. In our study, we evaluated CMI based on Th1-specific activation marker expression after *in vitro* re-stimulation of influenza-specific CD4+ and CD8+ T-cells. There were no CD8+ responses, but antigen-specific CD4+ responses were observed, expressing CD40L, IFN-γ, IL-2, and/or TNF-α, after primary and booster vaccination. CMI responses at after boosting at Month 6 appeared markedly higher in those primed with AS03_A_-adjuvanted vaccine than in those who received non-adjuvanted vaccine, supporting previous reports suggesting that adjuvantation has a positive priming effect on CD4+ T-cell responses.

The reactogenicity profile after booster vaccination was consistent with that observed during the primary vaccination study [13]. The most frequent injection site event was pain, and Grade 3 pain was uncommon, as were other local events such as redness, swelling, and ecchymosis. After booster vaccinations the most frequent general solicited events were myalgia, headache, and fatigue. The frequency of solicited symptoms observed after booster vaccination was consistent with that observed during the primary vaccination study [13]. During the 30-day period after booster vaccinations, ≤24.5% of subjects reported at least one unsolicited AE, and the rate of Grade 3 unsolicited AEs was low (≤2.8%). No safety concerns were identified, and the safety profile of the AS03_A_-H5N1 booster vaccine appeared acceptable.

## Conclusion

In conclusion, priming with two doses of AS03_A_-adjuvanted H5N1 pandemic influenza vaccine provides robust and durable, cross-clade immunogenicity. Strong cross-clade anamnestic responses were observed after one dose of AS03_A_-H5N1 heterologous booster vaccine given up to 36 months after priming, supporting the feasibility of highly flexible prime–boost schedules with AS03_A_-adjuvanted H5N1 vaccines. No safety concerns were raised during the 48 month extension study period.

*Prepandrix* is a trademark of the GlaxoSmithKline group of companies.

## Abbreviations

WHO: World Health Organisation; HA: Hemagglutinin antigen; HI: Hemagglutination inhibition; AEs: Adverse events; SAEs: Serious adverse events; GSK: GlaxoSmithKline; GMT: Geometric mean titer; SPR: Seroprotection rate; SCR: Seroconversion rate; CMI: Cell-mediated immunity; MedDRA: Medical Dictionary for Regulatory Activities; CI: Confidence interval; CBER: Center for Biologics Evaluation and Research.

## Competing interests

GlaxoSmithKline Biologicals SA was the funding source and was involved in all stages of the study conduct and analysis. GlaxoSmithKline Biologicals SA also took charge of all costs associated with the development and publishing of the manuscript. All authors had full access to the data, and the lead author had the final responsibility to submit the manuscript for publication.

PG, MD, KW, and FR are employees of GSK group of companies. PG, KW, and FR own GSK stock/stock options/shares. FSL reports receiving sponsorship to present at overseas conferences. DWSC, S-JH, P-CY, and PT declare no conflict of interest.

## Authors’ contributions

All authors participated in the design, implementation, analysis and interpretation of the study. All authors read and approved the final manuscript. DWSC, S-JH, P-CY, PT, and FSL were principal investigators. MD was responsible for the statistical analyses. KW contributed substantially to the analysis of immunological data. PG and FR were involved in all phases of the study, and are part of the clinical team at GSK.

## Pre-publication history

The pre-publication history for this paper can be accessed here:

http://www.biomedcentral.com/1471-2334/14/142/prepub

## Supplementary Material

Additional file 1 Cell-mediated immune responses against the booster vaccine strain (A/Indonesia/5/2005) primary vaccine strain (A/Vietnam/1194/2004) after heterologous booster vaccine at Month 6, 12, 36, and persistence of responses up to 48 months follow-up (cell-mediated immunity subset). Click here for file
