# Supplementary material for: Long-term booster schedules with AS03A-adjuvanted heterologous H5N1 vaccines induces rapid and broad immune responses in Asian adults
Source: BMC Infect Dis. 2014 Mar 15;14:142. doi: 10.1186/1471-2334-14-142 (PMC4008266; doi:10.1186/1471-2334-14-142)
Supplement: Additional file 1 — Cell-mediated immune responses against the booster vaccine strain (A/Indonesia/5/2005) primary vaccine strain (A/Vietnam/1194/2004) after heterologous booster vaccine at Month 6, 12, 36, and persistence of responses up to 48 months follow-up (cell-mediated immunity subset). [file 1471-2334-14-142-S1.docx]

**Additional file 1.**

**Cell-mediated immune responses against the booster vaccine strain (A/Indonesia/5/2005 ) primary vaccine strain (A/Vietnam/1194/2004) after heterologous booster vaccine at Month 6, 12, 36, and persistence of responses up to 48 months follow-up (cell-mediated immunity subset)**

| **Booster time-point** | **Sample time-point (month)** | **Influenza-specific CD4 T-cells expressed as median value per million CD4 T-cells (first quartile, third quartile)** | | | | | | | | | |
| --- | --- | --- | --- | --- | --- | --- | --- | --- | --- | --- | --- |
|  |  | **All doubles** | | **CD40L** | | **IFN-γ** | | **IL-2** | | **TNF-α** | |
|  |  | **Booster strain** | **Primary strain** | **Booster strain** | **Primary strain** | **Booster strain** | **Primary strain** | **Booster strain** | **Primary strain** | **Booster strain** | **Primary strain** |
| **Month 6** | 6 | n=2  909.0  (649.0, 1169.0) | n=2  1615.5  (918.0, 2313.0) | n=2  1045.0  (817.0, 1273.0) | n=2  1586.0  (857.0, 2315.0) | n=2  537.5  (431.0, 644.0) | n=2  826.0  (365.0, 1287.0) | n=2  883.5  (612.0, 1155.0) | n=2  1493.0  (952.0, 2034.0) | n=2  909.0  (649.0, 1169.0) | n=2  1280.5  (803.0, 1758.0) |
|  | 6+21 days | n=4  4249.5  (2151.5, 6892.0) | n=4  7067.5  (3359.5, 10769.5) | n=4  5292.0  (3027.0, 8110.0) | n=4  6931.0  (3256.0, 10610.5) | n=4  3255.0  (1676.0, 4878.5) | n=4  3608.5  (1467.5, 5510.5) | n=4  4972.5  (2846.0, 7782.5) | n=4  6557.0  (3110.5, 9871.0) | n=4  4249.5  (2151.5, 6892.0) | n=4  5527.5  (2298.0, 8633.5) |
|  | 12 | n=5  2528.0  (2107.0, 2752.0) | n=5  2410.0  (2288.0, 3872.0) | n=5  2634.0  (2382.0, 2917.0) | n=5  2384.0  (2257.0, 3832.0) | n=5  1217.0  (1046.0, 1782.0) | n=5  1108.0  (876.0, 2433.0) | n=5  2580.0  (2290.0, 2770.0) | n=5  2305.0  (2075.0, 3663.0) | n=5  2528.0  (2107.0, 2752.0) | n=5  1784.0  (1683.0, 3353.0) |
|  | 18 | n=5  1462.0  (1459.0, 1585.0) | n=5  2505.0  (2491.0, 2790.0) | n=5  2025.0  (1609.0, 2223.0) | n=5  2517.0  (2456.0, 2776.0) | n=5  989.0  (956.0, 1206.0) | n=5  1395.00  (1047.0, 1573.0) | n=5  1990.0  (1430.0, 2290.0) | n=5  2431.0  (2425.0, 2750.0) | n=5  1462.0  (1459.0, 1585.0) | n=5  2258.0  (2147.0, 2346.0) |
|  | 24 | n=4  2084.5  (1331.5, 2414.5) | n=4  2720.0  (1726.5, 3263.5) | n=4  2583.0  (1857.0, 3135.5) | n=4  2735.5  (1693.0, 3280.5) | n=4  1590.5  (1036.0, 1905.0) | n=4  1590.5  (1036.0, 1905.0) | n=4  2423.5  (1653.5, 2937.0) | n=4  2598.0  (1545.5, 3129.5) | n=4  2084.5  (1331.5, 2414.5) | n=4  1871.0  (1145.5, 2420.0) |
|  | 30 | n=2  2442.5  (2119.0, 2766.0) | n=2  2959.5  (2252.0, 3667.0) | n=2  3113.0  (2497.0, 3729.0) | n=2  2882.0  (2175.0, 3589.0) | n=2  1829.0  (1464.0, 2194.0) | n=2  1885.50  (1473.0, 2298.0) | n=2  2830.5  (2319.0, 3342.0) | n=2  2773.0  (2137.0, 3409.0) | n=2  2442.5  (2119.0, 2766.0) | n=2  2191.0  (1720.0, 2662.0) |
|  | 36 | n=4  1476.5  (697.5, 2289.0) | n=4  1420.0  (645.0, 2468.0) | n=4  1683.0  (848.0, 2642.5) | n=4  1406.0  (654.0, 2475.0) | n=4  1026.0  (481.0, 1548.5) | n=4  1026.0  (481.0, 1548.5) | n=4  1490.5  (770.0, 2396.0) | n=4  1301.0  (674.5, 2268.0) | n=4  1476.5  (697.5, 2289.0) | n=4  1145.0  (501.0, 1974.0) |
|  | 42 | n=5  1218.0  (558.0, 2068.0) | n=5  1354.0  (1190.0, 2571.0) | n=5  1656.0  (873.0, 2719.0) | n=5  1340.0  (1174.0, 2557.0) | n=5  698.0  (349.0, 1416.0) | n=5  547.0  (546.0, 1515.0) | n=5  1533.0  (801.0, 2472.0) | n=5  1218.0  (1125.0, 2362.0) | n=5  1218.0  (558.0, 2068.0) | n=5  1094.0  (818.0, 2000.0) |
|  | 48 | n=5  1169.0  (1028.0, 1376.0) | n=5  1600.0  (1374.0, 2493.0) | n=5  1496.0  (1252.0, 1799.0) | n=5  1618.0  (1387.0, 2449.0) | n=5  964.0  (721.0, 1157.0) | n=5  749.0  (697.0, 1682.0) | n=5  1373.0  (1115.0, 1814.0) | n=5  1169.0  (966.0, 2422.0) | n=5  1169.0  (1028.0, 1376.0) | n=5  1376.0  (1197.0, 1769.0) |
| **Month 12** | 6 | n=3  1644.0  (1334.0, 2617.0) | n=3  2133.0  (1392.0, 2727.0) | n=3  1792.0  (1691.0, 2986.0) | n=3  1998.0  (1335.0, 2496.0) | n=3  1189.0  (862.0, 1570.0) | n=3  999.0  (452.0, 1623.0) | n=3  1657.0  (1639.0, 2798.0) | n=3  1957.0  (1382.0, 2522.0) | n=3  1644.0  (1334.0, 2617.0) | n=3  1768.0  (1240.0, 1938.0) |
|  | 12 | n=7  1515.0  (1346.0, 1674.0) | n=7  1889.0  (1263.0, 2305.0) | n=7  1494.0  (1425.0, 1872.0) | n=7  1863.0  (1206.0, 2249.0) | n=7  994.0  (607.0, 1073.0) | n=7  884.0  (608.0, 925.0) | n=7  1649.0  (1293.0, 1865.0) | n=7  1729.0  (1286.0, 2197.0) | n=7  1515.0  (1346.0, 1674.0) | n=7  1488.0  (925.0, 1636.0) |
|  | 12+21 days | n=6  8466.0  (3885.0, 17018.0) | n=6  10061.5  (4553.0, 18155.0) | n=6  9416.0  (4129.0, 17348.0) | n=6  9587.0  (4451.0, 17713.0) | n=6  4661.5  (1676.0, 11231.0) | n=6  4918.0  (1852.0, 9857.0) | n=6  8993.0  (3979.0, 16403.0) | n=6  9252.0  (4333.0, 17247.0) | n=6  8466.0  (3885.0, 17018.0) | n=6  7385.5  (3438.0, 16134.0) |
|  | 18 | n=7  1810.0  (1451.0, 3453.0) | n=7  4073.0  (2335.0, 4833.0) | n=7  2240.0  (1692.0, 4454.0) | n=7  3980.0  (2294.0, 4803.0) | n=7  1306.0  (1049.0, 2032.0) | n=7  1927.0  (1061.0, 2642.0) | n=7  2297.0  (1694.0, 4071.0) | n=7  4073.0  (2281.0, 4495.0) | n=7  1810.0  (1451.0, 3453.0) | n=7  3357.0  (1879.0, 4452.0) |
|  | 24 | n=5  2972.0  (1281.0, 4109.0) | n=5  3945.0  (1666.0, 4592.0) | n=5  3435.0  (1801.0, 4486.0) | n=5  3932.0  (1667.0, 4539.0) | n=5  2036.0  (854.0, 2315.0) | n=5  2439.0  (613.0, 2625.0) | n=5  3256.0  (1653.0, 4370.0) | n=5  3799.0  (1480.0, 4419.0) | n=5  2972.0  (1281.0, 4109.0) | n=5  3252.0  (1107.0, 4049.0) |
|  | 30 | n=5  3247.0  (1042.0, 3614.0) | n=5  3713.0  (1996.0, 5220.0) | n=5  3652.0  (1296.0, 4172.0) | n=5  3607.0  (1908.0, 5200.0) | n=5  1892.0  (838.0, 2466.0) | n=5  2248.0  (1264.0, 2469.0) | n=5  3459.0  (1251.0, 4078.0) | n=5  3537.0  (1910.0, 5234.0) | n=5  3247.0  (1042.0, 3614.0) | n=5  3246.0  (1438.0, 4397.0) |
|  | 36 | n=7  2291.0  (951.0, 3523.0) | n=7  2149.0  (977.0, 4081.0) | n=7  2453.0  (958.0, 3867.0) | n=7  2122.0  (962.0, 4049.0) | n=7  1452.0  (606.0, 2023.0) | n=7  1260.0  (512.0, 2163.0) | n=7  2247.0  (1015.0, 3681.0) | n=7  2240.0  (798.0, 3945.0) | n=7  2291.0  (951.0, 3523.0) | n=7  1935.0  (820.0, 3658.0) |
|  | 42 | n=7  1901.0  (811.0, 2695.0) | n=7  2264.0  (1157.0, 3817.0) | n=7  2243.0  (1055.0, 3311.0) | n=7  2265.0  (1116.0, 3800.0) | n=7  1336.0  (568.0, 1805.0) | n=7  1218.0  (449.0, 2058.0) | n=7  2123.0  (1069.0, 3248.0) | n=7  2179.0  (1075.0, 3878.0) | n=7  1901.0  (811.0, 2695.0) | n=7  1819.0  (858.0, 3069.0) |
|  | 48 | n=6  1769.5  (1166.0, 3244.0) | n=6  2087.5  (1236.0, 4476.0) | n=6  2013.5  (1418.0, 3905.0) | n=6  2008.5  (1236.0, 4383.0) | n=6  1085.0  (787.0, 2692.0) | n=6  1007.0  (646.0, 2797.0) | n=6  1906.0  (1264.0, 3659.0) | n=6  1931.5  (1151.0, 3830.0) | n=6  1769.5  (1166.0, 3244.0) | n=6  1737.0  (941.0, 3622.0) |
| **Month 36** | 6 | n=4  2579.0  (2327.0, 2985.5) | n=8  3047.0  (1913.0, 3790.0) | n=4  2829.5  (2605.5, 3232.0) | n=8  2937.5  (1851.0, 3612.0) | n=4  2134.0  (1755.5, 2525.5) | n=4  1420.0  (948.0, 2423.0) | n=4  2799.0  (2495.5, 3049.0) | n=8  2910.5  (1768.5, 3368.5) | n=4  2579.0  (2327.0, 2985.5) | n=8  2540.0  (1451.5, 2899.0) |
|  | 12 | n=13  1174.0  (975.0, 2031.0) | n=14  1849.5  (1327.0, 2605.0) | n=13  1372.0  (1039.0, 1991.0) | n=14  1741.5  (1327.0, 2484.0) | n=13  852.0  (525.0, 1081.0) | n=13  869.50  (633.0, 1329.0) | n=13  1335.0  (961.0, 1859.0) | n=14  1759.0  (1393.0, 2469.0) | n=13  1174.0  (975.0, 2031.0) | n=14  1404.0  (1142.0, 2111.0) |
|  | 18 | n=15  1160.0  (894.0, 1492.0) | n=15  1658.0  (1301.0, 1927.0) | n=15  1518.0  (1148.0, 1929.0) | n=15  1607.0  (1274.0, 1819.0) | n=15  959.0  (678.0, 1151.0) | n=15  941.0  (548.0, 1263.0) | n=15  1224.0  (1164.0, 1695.0) | n=15  1592.0  (1235.0, 1833.0) | n=15  1160.0  (894.0, 1492.0) | n=15  1392. 0  (996.0, 1518.0) |
|  | 24 | n=13  1107.0  (1005.0, 1333.0) | n=13  1472.0  (1264.0, 1906.0) | n=13  1395.0  (1240.0, 1724.0) | n=13  1490.0  (1279.0, 1933.0) | n=13  787.0  (655.0, 947.0) | n=13  847.0  (658.0, 1112.0) | n=13  1187.0  (1107.0, 1534.0) | n=13  1454.0  (1093.0, 1693.0) | n=13  1107.0  (1005.0, 1333.0) | n=13  1131.0  (934.0, 1419.0) |
|  | 30 | n=13  1269.0  (1036.0, 1889.0) | n=13  1478.0  (1275.0, 2210.0) | n=13  1685.0  (1153.0, 2195.0) | n=13  1449.0  (1289.0, 2123.0) | n=13  1096.0  (758.0, 1330.0) | n=13  1074.0  (859.0, 1155.0) | n=13  1506.0  (1037.0, 2122.0) | n=13  1344.0  (1262.0, 2086.0) | n=13  1269.0  (1036.0, 1889.0) | n=13  1246.0  (987.0, 1662.0) |
|  | 36 | n=13  917.0  (640.0, 969.0) | n=13  1097.0  (836.0, 1395.0) | n=13  975.0  (849.0, 1278.0) | n=13  1098.0  (783.0, 1328.0) | n=13  543.0  (380.0, 662.0) | n=13  621.0  (526.0, 730.0) | n=13  846.0  (755.0, 1077.0) | n=13  891.0  (808.0, 1247.0) | n=13  917.0  (640.0, 969.0) | n=13  829.0  (569.0, 1071.0) |
|  | 36+21 days | n=12  3726.5  (3454.5, 5939.5) | n=12  5315.5  (4283.5, 7167.0) | n=12  5038.0  (4283.0, 7018.0) | n=12  5296.0  (4267.0, 7144.5) | n=12  2077.5  (1745.0, 2972.5) | n=12  2142.0  (1763.5, 2954.5) | n=12  4532.0  (4133.5, 6494.0) | n=12  4759.5  (3940.5, 6757.0) | n=12  3726.5  (3454.5, 5939.5) | n=12  3899.0  (3218.0, 6044.5) |
|  | 42 | n=15  1675.0  (1218.0, 2018.0) | n=15  2555.0  (1665.0, 2882.0) | n=15  2142.0  (1584.0, 2451.0) | n=15  2502.0  (1638.0, 2868.0) | n=15  1119.0  (714.0, 1728.0) | n=15  1228.0  (949.0, 1682.0) | n=15  2072.0  (1472.0, 2250.0) | n=15  2323.0  (1693.0, 2676.0) | n=15  1675.0  (1218.0, 2018.0) | n=15  1863.0  (1390.0, 2182.0) |
|  | 48 | n=13  1794.0  (1106.0, 2505.0) | n=13  2332.0  (1451.0, 3052.0) | n=13  2008.0  (1433.0, 2856.0) | n=13  2332.0  (1451.0, 2885.0) | n=13  1334.0  (553.0, 1822.0) | n=13  1223.0  (658.0, 1866.0) | n=13  1926.0  (1292.0, 2721.0) | n=13  2218.0  (1271.0, 2642.0) | n=13  1794.0  (1106.0, 2505.0) | n=13  1781.0  (1203.0, 2307.0) |
